# ID 167 - Cenário Atual dos Métodos para Dimensionamento Corporal para Fabricação de OPM não Implantáveis: uma revisão de escopo

**DOI:** 10.5327/2237-9622.2025.v34s1.167

**Published:** 2025-11-25

**Authors:** Ketinlly Yasmyne Nascimento Martins, Hélder Xavier Bezerra, Rodolfo Ramos Castelo Branco, Ana Cheile de Melo Costa, Ana Carolina Rodrigues Alves, Kátia Elizabete Galdino, Eduardo Jorge Valadates Oliveira

**Keywords:** aparelhos ortopédicos, equipamentos de autoajuda, produção de produtos, tecnologias de produtos, processos de fabricação

## Abstract

**Introdução:**

No Brasil, onde cerca de 23,9% da população têm alguma deficiência, o acesso às órteses, próteses e meios auxiliares de locomoção (OPM) é limitado devido à fabricação inadequada e à falta de personalização. No processo de fabricação desses dispositivos, uma das fases mais importantes é a análise do dimensionamento corporal, pois eles devem se adaptar perfeitamente ao corpo para evitar desconfortos e abandono. Nesse sentido, o presente estudo tem como objetivo mapear os conceitos e as evidências existentes na literatura sobre os métodos de dimensionamento corporal existentes para fabricação de OPMs não relacionadas ao ato cirúrgico.

**Método:**

Estudo do tipo revisão de escopo. A pergunta norteadora foi estruturada pelo acrônimo População, Conceito e Contexto (PCC) (Quais os diferentes métodos existentes na literatura de dimensionamento corporal para fabricação de OPME?). Foram incluídos estudos que apresentassem ao menos um método de dimensionamento corporal e documentos oficiais de entidades governamentais e não governamentais, sem recorte temporal e sem restrições de idioma. E excluídos os estudos que não estivessem disponíveis na íntegra, bem como os que apresentassem métodos relacionados ao ato cirúrgico. As buscas foram realizadas em cinco bases de dados eletrônicas (LILACS, Embase, MEDLINE via PubMed, Cochrane Library e Scopus), além da literatura cinzenta. A seleção dos estudos ocorreu em duas etapas: triagem de títulos e resumos, seguida de análise dos textos completos com base nos critérios de elegibilidade.

**Resultados:**

No total, 1.267 estudos foram identificados, dos quais 27 foram excluídos por serem duplicatas. Após a triagem por título e resumo, 86 estudos foram selecionados para leitura completa, resultando na exclusão de 43 e na inclusão de 43 estudos na revisão. Os estudos incluídos foram publicados entre 2005 e setembro de 2022, com a maioria (51,1%) entre 2016 e 2020, e todos foram artigos de periódicos científicos. A maioria dos estudos (90,7%) eram estudos de caso, enquanto 7% eram ensaios clínicos e 2,3% revisões. Os métodos de dimensionamento mais comuns foram a digitalização 3D (81,3%) e o molde de gesso (23,3%). A tomografia computadorizada (13,9%) e radiografias biplanares (4,6%) foram menos comuns. Os dispositivos mais frequentemente desenvolvidos foram órteses, com predominância de órteses de membro inferior (34,8%) e de tronco (20,9%).

**Conclusão:**

Embora técnicas tradicionais como o uso de moldes de gesso ainda sejam comuns devido às habilidades especializadas necessárias, elas apresentam limitações significativas, incluindo desconforto para o paciente e ineficiências na fabricação. Em contraste, métodos modernos como digitalização 3D, tomografia computadorizada e radiografias biplanares oferecem avanços em precisão e eficiência, melhorando o ajuste e o conforto dos dispositivos ortopédicos e protéticos. A digitalização 3D, por exemplo, reduz o tempo de fabricação, embora o custo inicial elevado e a necessidade de treinamento especializado ainda sejam desafios a serem superados. Além disso, a resistência à mudança por parte dos profissionais e a necessidade de maior investimento em infraestrutura podem limitar a adoção dessas tecnologias. As conclusões deste estudo demonstram a evolução dos métodos de dimensionamento corporal e o impacto potencial na qualidade das OPMs. A implementação bem-sucedida dessas tecnologias poderá melhorar substancialmente a fabricação de dispositivos ortopédicos e protéticos, beneficiando tanto os pacientes quanto os profissionais da área.

